# Retraction: Interferon and Ribavirin Combination Treatment Synergistically Inhibit HCV Internal Ribosome Entry Site Mediated Translation at the Level of Polyribosome Formation

**DOI:** 10.1371/journal.pone.0308579

**Published:** 2024-08-05

**Authors:** The PLOS ONE Editors

After publication of the Expression of Concern on this article [1, 2], additional concerns were raised about results presented in Figs 2 and 7. Specifically:

- The EGFPN1 GFP RBV panel in Fig 2A appears similar to the eIF2α RBV panel in Fig 7A.
- In Fig 7A:
  ○ The PKR IF-N-α panel appears similar to the eIF2α IF-N-α panel.
  ○ The PKR RBV panel appears similar to the IMPDH RBV panel.

In response to the concerns listed above and in [2], the corresponding author stated that the underlying data for the experiments in question are no longer available.

In light of the extent of the concerns listed above and in [2] that cannot be resolved in the absence of the original underlying data and which question the reliability of the reported results and conclusions, the *PLOS ONE* Editors retract this article.

RFG agreed with the retraction. RP, SH, SC, PKC, SDatta, RK, CEC, ZH, HZ, and LAB either did not respond directly or could not be reached. SDash did not agree with the retraction and stands by the article’s findings.

## References

[1] Panigrahi R, Hazari S, Chandra S, Chandra PK, Datta S, Kurt R, et al. (2013) Interferon and Ribavirin Combination Treatment Synergistically Inhibit HCV Internal Ribosome Entry Site Mediated Translation at the Level of Polyribosome Formation. PLoS ONE 8(8): e72791. 10.1371/journal.pone.0072791 24009705 PMC3751885

[2] The *PLOS ONE* Editors (2022) Expression of Concern: Interferon and Ribavirin Combination Treatment Synergistically Inhibit HCV Internal Ribosome Entry Site Mediated Translation at the Level of Polyribosome Formation. PLoS ONE 17(3): e0266498. 10.1371/journal.pone.0266498 35358293 PMC8970492

